# Mean platelet volume can indicate dietary adherence and disease severity of celiac disease

**DOI:** 10.14744/nci.2021.56313

**Published:** 2022-02-11

**Authors:** Emre Gerceker, Ahmed Ramiz Baykan, Serkan Cerrah, Hakan Yuceyar

**Affiliations:** 1. Department of Gastroenterology, Gazi Hospital, Izmir, Turkey; 2. Department of Gastroenterology, Erzurum Regional Training and Research Hospital, Erzurum, Turkey; 3. Department of Gastroenterology, Celal Bayar University Faculty of Medicine, Manisa, Turkey

**Keywords:** Celiac disease, dietary adherence, mean platelet volume

## Abstract

**Objective:**

At present, there is no reliable indicator for dietary compliance and disease severity in patients with celiac disease (CD). The aim of this study is to evaluate mean platelet volume (MPV) level as a biomarker for detection of disease activation, dietary adherence, and assessment of disease severity.

**Methods:**

Eighty-one patients with CD and 50 healthy subjects were enrolled in this study. The diagnosis of CD was established by both positive antibodies against endomysium or gliadin and histopathological criteria (lymphocytic inﬁltration and total villous atrophy in duodenal biopsies).

**Results:**

MPV was observed to be significantly higher among CD patients when compared to healthy controls (8.14±0.26 fL vs. 7.82±0.29 fL and p=0.001). Overall dietary adherence rate was 72.8% (58/81 CD patients). After induction of a gluten-free diet, the MPV was signiﬁcantly lower in the dietary adherent group than non-adherent patients (7.86±0.17 fL vs. 8.07±0.30 fL and p=0.001). The increase of MPV was correlated with Marsh classification (Marsh 3 active CD vs. Marsh 2 active CD vs. Marsh 1 active CD; 8.32±0.27 fL vs. 8.12±0.19 fL vs. 7.98±0.19 fL; p=0.004 and p=0.009).

**Conclusion:**

Based on these data, we believe that increased MPV can provide additional benefit to screening in patients with CD. It can indicate the activation of the disease and adherence to the diet.

**C**eliac disease (CD) is an autoimmune disorder that affects genetically predetermined individuals, causing underlying sensitivity to gluten. Predominant pathology in CD is progressive inflammation of the small intestine. This inflammation ultimately results in malabsorption, unless gluten-containing food is restricted [1]. Removing gluten from the diet is the main therapeutic approach that can achieve significant clinical and serologic improvement in most patients with CD [2]. The diagnosis of CD requires the presence of typical clinical symptoms, detection of serum antibodies against endomysium or gliadin and the presence of histopathological Marsh criteria (including pathognomonic findings, e.g., the presence of intraepithelial lymphocytes, villus atrophy, and crypt hyperplasia) [3]. Monitoring of antibody titers and evaluation of mucosal biopsy have some limitations during daily clinical practice. At present, none of the markers are reliable indicators for dietary adherence and disease severity in patients with CD. A group of patients present with mild clinical and laboratory findings that may lead to delayed diagnosis [3–5]. Early diagnosis is important to prevent the development of CD-related complications (Anemia, vitamin and mineral disorders, osteopenia, osteoporosis, malabsorption, and development of T-cell lymphoma) [1, 2, 6]. Mean platelet volume (MPV) has recently been recognized as an important inflammatory marker in a variety of chronic inflammatory diseases, including ulcerative colitis, Crohn’s disease, and connective tissue diseases. Although some studies show negative correlation between MPV and disease activity, various researchers have reported a significant relation between MPV and disease severity [7]. Correlation between MPV and CD was first reported by Oberhuber [8] Purnak et al. [9] reported MPV as a biomarker for monitoring dietary adherence over time in patients with CD. The aim of this study is to investigate the value of MPV and platelet level as biomarkers for detection of disease activation, dietary adherence, and also assessment of disease severity.

## Materials and Methods

This study was carried out applying good clinical practice to comply with the Helsinki Declaration. Ethical approval (#2019/16-149) for the study was obtained from the Institutional Ethical Review Board of Erzurum Regional Training and Research Hospital on the December 16, 2019. Celiac patients diagnosed at the Gastroenterology Department of Erzurum Regional Training and Research Hospital between January 2014 and December 2018 were enrolled in the study. Cases with symptoms of CD, positive antibodies to gliadin and/or endomysium, typical findings of CD on endoscopic evaluation and positive histopathological findings of CD according to the modified Marsh classification in duodenal mucosal biopsies were considered to be active celiac patients [1, 3]. Hemogram, routine biochemical examinations, endoscopic findings, ultrasound findings, histopathological evaluation findings in duodenal biopsy and Marsh classification, anti-gliadin, and endomysium antibody levels were retrospectively scanned and recorded in all patients with CD.

Dietary compliance was questioned and recorded during first control visit, 3 months after the initiation of gluten-free celiac diet therapy. Patients, who did not show any signs of CD, had complete dietary adherence, normal endoscopic appearance in the duodenal mucosa at endoscopic controls, and normal pathological findings of mucosal biopsies were considered to be in remission and diet-adherent CD at the 3^rd^ month follow-up. Patients that showed signs of CD, did not have or did not full complete with diet, had findings of CD in the duodenal mucosa at endoscopic controls and/or were found to have CD findings in the pathological evaluation of mucosal biopsies were defined as dietary non-adherent and non-remitting CD at the 3^rd^ month follow-up [1, 3].

Hemogram and biochemical examinations performed during the control period among both dietary adherent and non-adherent patients (patients without remission) were also retrospectively scanned and recorded.

Patients with functional gastrointestinal disorders that had normal complete blood count, erythrocyte sedimentation rate (ESR), serum C-reactive protein (CRP) levels, negative for antibodies to gliadin and endomysium, and normal findings in the upper gastrointestinal endoscopy performed for dyspepsia were chosen to form the control group. Hemogram, routine biochemical examinations, endoscopic findings, ultrasound findings, histopathological findings of duodenal biopsies, and anti-gliadin and endomysium antibody levels of the control group were retrospectively scanned and recorded.

Patients with a diagnosis of heart failure, peripheral vascular disease, acute or chronic infection, cancer, hematological, and hepatic disorders were excluded from the study. Patients that are on chronic medications (nonsteroidal anti-inflammatory drugs, anticoagulants, and oral contraceptives) were excluded from the study.

### Statistical Analysis

Statistical assessment was made using “SPSS 22 for Windows” software package. Categorical (nominal) values are expressed as percentage (%) and compared with the Chi-square test (χ^2^). Continuous numerical (quantitative) values were expressed as mean±standard deviation. Quantitative variables were compared with “Student t-test” and “ANOVA,” if p<0.05 was determined as statistically significant.

## Results

A total of 131 subjects including 81 patients with CD and 50 controls were included in the study. About 33.3% (n=27) of patients with CD had Marsh 3 activation, 32.1% (n=26) had Marsh 2 activation, and 34.6% (n=28) had Marsh 1 activation. The mean age of all cases was 37.69±10.65 (19–74). About 64.1% of the patients were female. Table 1 summarizes demographic and clinical findings of the patients with CD and the control group. Mean age, gender ratio, Aspartate transaminase, Alanine transaminase albumin levels, and spleen size of the both groups were similar.

**Table 1.** Demographic features of the patients with CD and healthy controls

	Patients with CD	Control group	p
Age (years)	38.74±10.66	35.98±10.50	0.584
Gender (female) (%)	65.4	62	0.822
Hemoglobin (mg/L)	12.73±1.53	13.44±0.80	0.481
AST (IU)	23.44±19.23	25.50±8.03	0.796
ALT (IU)	20.21±14.27	20.58±8.95	0.343
Albumin (mg/L)	3.52±0.53	3.54±0.57	0.308
Spleen size (mm)	93.19±5.55	93.18±5.51	0.790
Dietary adherence (%)	72.8

CD: Celiac disease; AST: Aspartate transaminase; ALT: Alanine transaminase.

Inflammatory markers of the CD patients with activation and control groups are summarized in Table 2. Platelet levels were signiﬁcantly higher in CD group with activation when compared to healthy controls (294.48±28.40 vs. 261.10±28.29 and p=0.001). MPV was signiﬁcantly higher in the CD group with activation compared to healthy controls (8.14±0.26 vs. 7.82±0.29 and p=0.001). Other inflammatory biomarkers such as white blood cell (WBC), ESR, and CRP were similar between the CD patients with activation and control groups.

**Table 2.** Inflammatory markers of the CD patients with activation and control groups

	CD with activation	Control group	p
WBC (×10^9^/L)	7.48±2.21	7.34±1.83	0.707
ESR (mm/h)	12.19±2.92	12.34±2.77	0.782
CRP (mg/L)	2.93±1.60	2.79±1.65	0.613
Platelet (×10^9^/L)	294.48±28.40	261.10±28.29	0.001
MPV (fL)	8.14±0.26	7.82±0.29	0.001

CD: Celiac disease; CRP: C-reactive protein; ESR: Erythrocyte sedimentation rate; MPV: Mean platelet volume; WBC: White blood cell.

Inflammatory markers of the CD patients with activation and CD patients with remission are summarized in Table 3. Platelet levels were signiﬁcantly higher in the CD patients with activation compared to CD patients group with remission (294.48±28.40 vs. 262.51±20.80 and p=0.001). MPV levels were signiﬁcantly higher in the CD group with activation compared to CD patients group with remission (8.14±0.26 vs. 7.86±0.17 and p=0.001). Other inflammatory biomarkers such as WBC, ESR, and CRP were similar between CD patients with activation and CD patients with remission.

**Table 3.** Inflammatory markers of the CD patients with activation and CD patients with remission

	CD with activation	CD with remission	p
WBC (×10^9^/L)	7.48±2.21	7.27±1.85	0.554
ESR (mm/h)	12.19±2.92	12.08±2.67	0.815
CRP (mg/L)	2.93±1.60	2.95±1.64	0.960
Platelet (×10^9^/L)	294.48±28.40	262.51±20.80	0.001
MPV (fL)	8.14±0.26	7.86±0.17	0.001

CD: Celiac disease; CRP: C-reactive protein; ESR: Erythrocyte sedimentation rate; MPV: Mean platelet volume; WBC: White blood cell.

**Figure 1.**
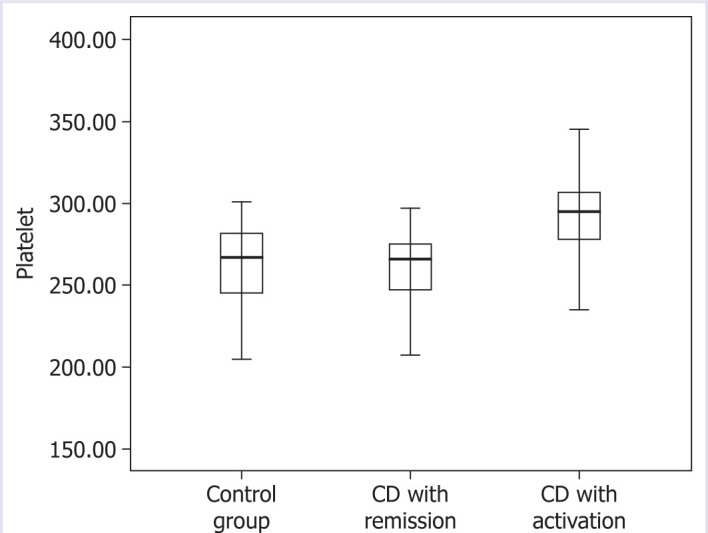
Comparison of platelet levels according to dietary adherence.

**Figure 2.**
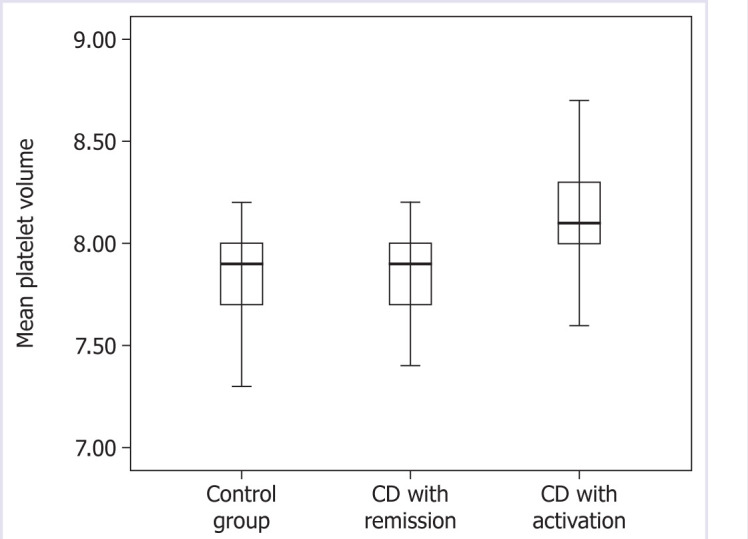
Comparison of MPV levels according to dietary adherence.

Inflammatory markers of dietary adherent and non-adherent patients with CD are summarized in Table 4, Figures 1 and 2. Platelet levels were signiﬁcantly lower in dietary adherent group compared to CD patients in non-adherent group (262.51±20.80 vs. 296.91±37.93 and p=0.001). MPV levels were signiﬁcantly lower in CD patients with dietary adherent group compared to CD patients in non-adherent group (7.86±0.17 vs. 8.07±0.30 and p=0.001). Other inflammatory biomarkers such as WBC, ESR, and CRP were similar between CD patients with dietary adherent group and non-adherent group.

**Table 4.** Inflammatory markers of the CD patients with dietary adherent and non-adherent group

	Dietary adherent	Dietary non-adherent	p
WBC (×10^9^/L)	7.27±1.85	7.50±1.84	0.621
ESR (mm/h)	12.08±2.67	11.27±3.04	0.245
CRP (mg/L)	2.95±1.64	3.06±1.51	0.794
Platelet (×10^9^/L)	262.51±20.80	296.91±37.93	0.001
MPV (fL)	7.86±0.17	8.07±0.30	0.001

CD: Celiac disease; CRP: C-reactive protein; ESR: Erythrocyte sedimentation rate; MPV: Mean platelet volume; WBC: White blood cell.

**Figure 3.**
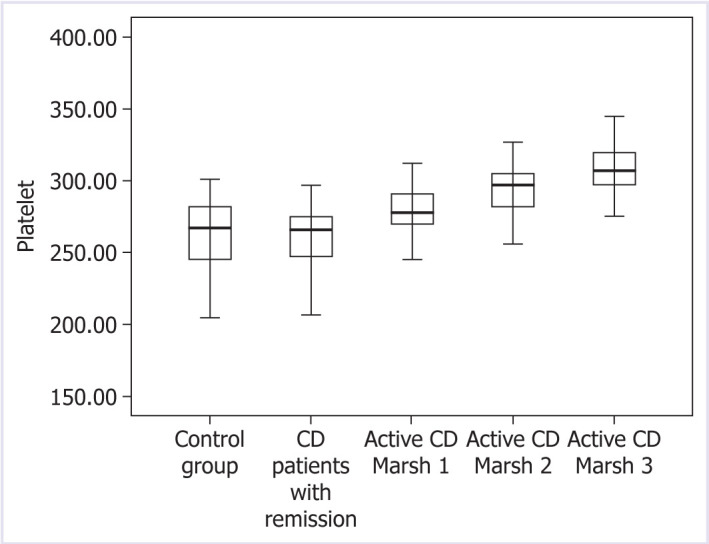
Comparison of platelet values according to disease activity and Marsh classification.

Platelet levels were signiﬁcantly higher in the Marsh 3 active CD group compared to Marsh 2 group (313.33±29.25 vs. 293.50±21.77 and p=0.007). Platelet levels were signiﬁcantly higher in the Marsh 2 active CD group compared to Marsh 1 group (293.50±21.77 vs. 277.21±21.50 and p=0.008). Platelet levels were signiﬁcantly higher in the Marsh 1 active CD group compared to CD patients group with remission (277.21±21.50 vs. 262.51±20.80 and p=0.003). Comparison of the platelet level according the Marsh classification is summarized in Figure 3.

**Figure 4.**
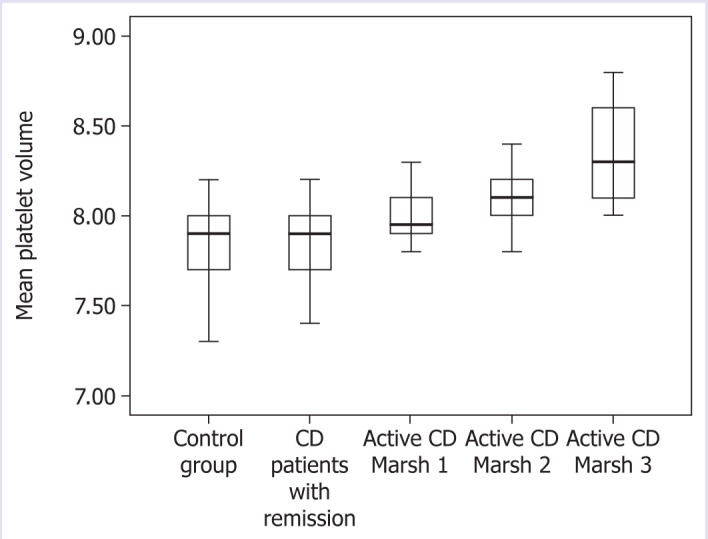
Comparison of MPV values according to disease activity and Marsh classification.

MPV levels were signiﬁcantly higher in the Marsh 3 active CD group compared to Marsh 2 active CD group (8.32±0.27 vs. 8.12±0.19 and p=0.004). MPV levels were signiﬁcantly higher in the Marsh 2 active CD group compared to Marsh 1 group (8.12±0.19 vs. 7.98±0.19 and p=0.009). MPV levels were signiﬁcantly higher in the Marsh 1 active CD group compared to CD patients group with remission (7.98±0.19 vs. 7.86±0.17 and p=0.003). Comparison of MPV levels according the Marsh classification is summarized in Figure 4.

## Discussion

In this study, MPV and platelet levels were signiﬁcantly higher in CD group with activation compared to healthy controls or patients with CD group in remission (defined as without CD symptoms, with normal mucosal findings at endoscopic controls, and with complete dietary compliance). Increased MPV and platelet levels observed in our study group of newly diagnosed CD patients may be due to on-going intestinal inﬂammation. On the other hand, normalization of MPV levels and decrease in platelet levels following gluten-free diet may indicate regression of intestinal inﬂammation. In our study, we observed that MPV and platelet levels increased in correlation with disease severity according to the Marsh classification. MPV and platelet levels were similar in the CD patients with remission, who had dietary compliance and normal mucosal findings in endoscopy and normal pathological findings in duodenal biopsies and the healthy controls. This is the first study to show the correlation of increased MPV and platelet levels with CD severity according to Marsh classification. Based on these data, we can suggest that elevated platelet level and MPV can provide additional benefit to screening in patients with CD. It can indicate that the activation of the disease and dietary compliance of patients with CD.

CD is a chronic auto-inﬂammatory disorder and requires life-long treatment and follow-up. Early diagnosis is important to prevent the development of complications associated with CD [1, 2]. Although we understand new aspects of the disease, the current treatment for CD is still gluten-free diet [10]. Monitoring dietary compliance is usually based on the patient’s own statement. There are few objective criteria for assessing dietary compliance among patients with CD. Histological improvement is the generally accepted gold standard to indicate dietary compliance and disease remission. However, this is an invasive and non-practical method for routine follow-up of patients with CD [11]. Although antibody titers against tissue transglutaminase, endomysium and gliadin are considered to be good indicators of remission in CD patients, they have some limitations in daily practice [12, 13]. Clinical and laboratory findings are not always sufficient for early diagnosis [4, 5]. At present, none of the available markers are reliable and accepted indicators for dietary compliance and disease severity in patients with CD. Studies indicate that platelet levels and MPV can provide important information on the course and prognosis in many inflammatory disorders such as ulcerative colitis, Crohn’s disease, rheumatoid arthritis, and juvenile systemic lupus erythematous [7]. There is only one study in the literature that investigated the relationship between MPV level and CD. Similar to our study, Purnak et al. [9] suggest that MPV can be a promising and easily available biomarker for monitoring dietary adherence in CD patients at a relatively lower cost. In this study, MPV and platelet levels were found to be higher in active celiac patients than in the control group. In the post-diagnosis controls, after the development of remission with gluten-free diet, decrease in MPV and platelet levels was observed [9].

Studies in the literature stated that MPV and platelet levels correlate with inflammatory disease severity other than CD [7]. There is no study in the literature showing the correlation between increased MPV or platelet levels and CD severity according to Marsh classification. Liu et al. [14] observed increased levels of MPV in patients with Crohn’s disease when compared to healthy subjects. Zubcevic et al. [15] indicated that elevated MPV levels can be a marker of disease activity in Crohn’s disease. Gasparyan et al. reported that increased MPV is observed due to administration of anti-inﬂammatory drugs in rheumatoid arthritis [16, 17]. Yavuz and Ece reported that increased MPV levels were associated with disease progression in patients diagnosed with juvenile SLE [18]. Yuksel et al. [19] also showed that decreased MPV levels were associated with increased activity of ulcerative colitis. Gasparyan et al. and Delgado-García et al. [16, 20] observed that decreased MPV levels were associated with disease exacerbation in rheumatoid arthritis. It has been shown that interleukin (IL)-6, IL-1, and TNF-α can stimulate precursor cells of platelets under inflammatory conditions [21]. The effect of IL-6 is related with enhanced thrombopoietin (TPO) production in the liver and its direct effect on megakaryocytes through the membranous receptor IL-6 receptor. As a result platelet count can increase significantly in an inflamed state. In ongoing inflammation, the increase in pro-inflammatory cytokines such as IL-6 stimulates TPO formation that leads to an increase in the ploidy of megakaryocyte nuclei and cytoplasm volume. As a result, more and larger platelet (elevated MPV) formation is observed [22]. According to the data of our study and other studies in the literature, we think that there is a positive correlation between the severity of inflammation in CD and increased MPV or platelet levels.

### Conclusion

The aim of our study was to investigate the value of MPV and platelet levels as biomarkers in the detection of disease activation, dietary adherence, and also assessment of disease severity in CD. Based on these data, we believe that elevated platelet level and MPV can provide additional benefit to screening in patients with CD. It can indicate that the activation of the disease and dietary compliance of patients with CD.
